# Adult Leigh Syndrome Associated with the m.15635T>C Mitochondrial DNA Variant Affecting the Cytochrome *b* (*MT-CYB*) Gene

**DOI:** 10.3390/ijms26031116

**Published:** 2025-01-27

**Authors:** Concetta Valentina Tropeano, Chiara La Morgia, Alessandro Achilli, Luisa Iommarini, Gaia Tioli, Leonardo Caporali, Anna Olivieri, Maria Lucia Valentino, Rocco Liguori, Piero Barboni, Andrea Martinuzzi, Caterina Tonon, Raffaele Lodi, Antonio Torroni, Valerio Carelli, Anna Maria Ghelli

**Affiliations:** 1 Programma di Neurogenetica, IRCCS Istituto delle Scienze Neurologiche di Bologna, 40139 Bologna, Italy; 2 Department of Biomedical and NeuroMotor Sciences (DIBINEM), University of Bologna, 40138 Bologna, Italy; 3 Department of Biology and Biotechnology “L. Spallanzani”, University of Pavia, 27100 Pavia, Italy; 4 Department of Pharmacy and Biotechnology (FABIT), University of Bologna, 40126 Bologna, Italy; 5 Studio Oculistico d’Azeglio, 40123 Bologna, Italy; 6 Department of Public Health and Pediatric Sciences, University of Torino, 10126 Torino, Italy; 7 Programma Neuroimmagini Funzionali e Molecolari, IRCCS Istituto delle Scienze Neurologiche di Bologna, 40139 Bologna, Italy

**Keywords:** Respiratory complex III, mtDNA, Leigh syndrome, *MT-CYB*

## Abstract

We report on a sporadic patient suffering Leigh syndrome characterized by bilateral lesions in the lenticular nuclei and spastic dystonia, intellectual disability, sensorineural deafness, hypertrophic cardiomyopathy, exercise intolerance, and retinitis pigmentosa. Complete sequencing of mitochondrial DNA revealed the heteroplasmic nucleotide change m.15635T>C affecting a highly conserved amino acid position (p.Ser297Pro) in the cytochrome *b* (*MT-CYB*) gene on a haplogroup K1c1a background, which includes a set of four non-synonymous polymorphisms also present in the same gene. Biochemical studies documented respiratory chain impairment due to complex III defect. This variant fulfils the criteria for being pathogenic and was previously reported in a sporadic case of fatal neonatal polyvisceral failure.

## 1. Introduction

Leigh syndrome is a severe encephalopathy hallmarked by bilateral subacute necrosis in the central nervous system (CNS) [1]. The bilateral lesions may have variable distribution, classically affecting the basal ganglia and brainstem. Most commonly, these lesions affect the CNS early in life, literally from the first days after birth to adolescence; more rarely, bilateral striatal necrosis may occur in adult life. A long list of more than 100 genetic causes has been identified to date [2,3]. Defects in virtually any gene leading to severe impairment of oxidative phosphorylation (OXPHOS) may underlie Leigh syndrome, affecting either nuclear-encoded (nDNA) genes as well as the mitochondrial DNA (mtDNA) genes [4]. However, while complex I and IV deficiencies are the most commonly associated with Leigh syndrome and Leigh-like phenotypes, isolated complex III defects have occasionally been reported, and mostly in relation to nDNA genes [5].

Complex III (CIII) defects due to mtDNA mutations affecting the cytochrome *b* (*MT-CYB*) gene, the only subunit encoded by mtDNA, are rare disorders of the respiratory chain [6,7]. In most cases, the phenotype is characterized by exercise intolerance and pure myopathy. Many of the *MT-CYB* mutations have been shown to be confined to skeletal muscle, possibly arising as early somatic mutational events [8]. However, the phenotypic spectrum of *MT-CYB* mutations has more recently widened, displaying some peculiarities. For example, unlike any of the other 12 mtDNA genes encoding respiratory chain complex subunits, the *MT-CYB* gene hosts a few micro-rearrangements leading to either in-frame shorter proteins [9] or truncated proteins due to frameshift and downstream stop codons [10,11]. Another noticeable feature of some *MT-CYB* mutations is the pathogenic mechanism implying complex I (CI) dysfunction through unstable CI-III supercomplex interaction, indicating a main role of CIII as a structural and functional platform for the overall respiratory chain biogenesis [7,12]. Consequently, *MT-CYB* mutations have been associated with multisystem phenotypes including severe CNS involvement, frequently resembling mitochondrial encephalomyopathy, lactic acidosis, and stroke-like syndrome (MELAS) [9,10,13,14,15].

Here, we report for the second time the occurrence of the m.15635T>C (p.Ser297Pro) variant affecting the *MT-CYB* gene in a patient with Leigh syndrome, previously found in a very severe sporadic case of fatal neonatal polyvisceral failure [16]. Investigation of patient-derived cybrids further consolidates the evidence for the pathogenic potential of this rare *MT-CYB* variant.

## 2. Materials and Methods

### 2.1. Genetic Investigations

Sequence analysis of mtDNA was carried out as previously reported [17]. Heteroplasmy was assessed by Next Generation Sequencing (NGS) approach, amplifying the region containing the *MT-CYB* variant with appropriate primers (available upon request). The library was constructed using xGen™ DNA Library Prep EZ (Integrated DNA Technologies, Coralville, IA, USA) on purified PCR products and 2 × 150 paired-end reads sequenced on a MiSeq System (Illumina, San Diego, CA, USA). The FASTQ files were analyzed with the combination of the mutserve, mitoscape, and Mutect2 pipelines.

### 2.2. Materials for Biochemical Assays

2,3-Dimethoxy-5-methyl-6-decyl-1,4-benzoquinone (DB), 3-(4,5-dimethyl thiazol-2yl)-2,5-diphenyl tetrazolium bromide (MTT), 5,5-dithio-bis-(2-nitrobenzoic acid) (DTNB), acetyl-CoA, adenosine diphosphate (ADP), aminocaproic acid, antimycin A, adenosine triphosphate (ATP), Bis-Tris, bovine serum albumin (BSA), cytochrome *c*, digitonin, EDTA, erythrosine B, galactose, glycerol, glycerol-3-phosphate (G3P), HEPES, K-acetate, KCl, KCN, malate, malonate, MgCl_2_, NaCl, NADH, oligomycin, oxaloacetate, P1-P5-di(adenosine-5′) pentaphosphate pentasodium salt, phenylmethanesulfonyl fluoride (PMSF), Ponceau S, potassium phosphate, sodium pyruvate, rotenone, Serva G Blue, succinate, sucrose, sulforhodamine B (SRB), trichloroacetic acid (TCA), Tris-HCl, triton X-100, and the ATP monitoring kit were purchased from Sigma (Milan, Italy). Primary antibodies against CI (NDUFA9, NDUFB8, and NDUFS3 subunits: cat. Ab14713, cat. Ab110242, and cat. Ab110246, respectively), CIII (UQCRC2 subunit, cat. Ab14745), complex IV (CIV) (COX-IV subunit, cat. Ab14744), and complex V (CV) (αATPase subunit, cat Ab14748) were obtained from Abcam, Cambridge, UK. The antibody against complex II (CII) (SDHA subunit cat. 459200) was obtained from Life Technologies, Milan, Italy. Horseradish peroxidase-conjugated secondary antibodies were obtained from Jackson ImmunoResearch, West Grove, PA, USA.

### 2.3. Cell Lines and Culture Conditions

Skin fibroblasts were established, following informed consent, from the proband’s mother’s skin biopsy carrying the m.15635T>C variant in the *MT-CYB* gene (~50% mutant load), leading to missense change p.Ser297Pro in the protein. Cybrid cell lines were generated using enucleated skin fibroblasts as mitochondria donors and the mtDNA-deprived human osteosarcoma 143B.TK cell line (Rho0 cells) as an acceptor, following the protocols described in [18]. After the selection of cybrid clones, a wild-type and a homoplasmic mutant cell line were obtained. Cybrids with wild-type mtDNA and skin fibroblasts were grown in Dulbecco’s modified Eagle medium (DMEM) supplemented with 10% fetal calf serum (South American source from Gibco, Life Technologies Italia, Milan, Italy), 2 mM L-glutamine, 100 U/mL penicillin, and 100 mg/mL streptomycin, in an incubator with a humidified atmosphere of 5% CO_2_ at 37 °C. Homoplasmic mutant cybrids were grown in the same conditions, adding 50 µg/mL uridine, being heterotrophs for pyrimidine synthesis. In some experiments, cells were incubated with a glucose-free DMEM supplemented with 5 mM galactose, 5 mM sodium pyruvate, and 5% fetal calf serum (DMEM–galactose).

### 2.4. Cell Viability

Cybrids were seeded (4 × 10^4^ cells/cm^2^) in 24-well plates and incubated with DMEM or with DMEM–galactose. At the times indicated, cell viability was measured by the SRB absorbance with a VICTOR3 Multilabel Plate Counter (PerkinElmer Life and Analytical Sciences, Zaventem, Belgium), as described in [19]. Briefly, the plates were incubated with 10% TCA for 1 h at 4 °C to fix the cells. Five washes in water were carried out. Once the plates were dried, proteins were stained by incubation with 0.4% SRB in 1% acetic acid for 30 min at RT, followed by four washes with 1% acetic acid. Then, SRB was solubilized with 10 mM Tris, and the absorbance at 570 nm was determined.

### 2.5. Citrate Synthase Activity

The activity of citrate synthase (CS) was evaluated in mitochondria suspended in a buffer containing 125 mM Tris-HCl pH 8, 0.1% Triton X-100, 100 μM DTNB (ε = 13.6 mM^−1^), and 300 μM acetyl-CoA. The reaction was started by the addition of 500 μM oxaloacetate [20] and measured at 412 nm at 30 °C with a UV-Vis spectrophotometer (V550 Jasco Europe, Modena, Italy).

### 2.6. Complex III Redox Activity Measurement

Redox enzymatic activity was measured in crude mitochondria obtained by harvesting approximately 15 × 10^6^ cells in phosphate-buffered saline (PBS), essentially as described in [21]. Briefly, after centrifugation, the pellet was suspended in the mitochondrial isolation buffer containing 0.25 M sucrose, 10 mM Tris-HCl pH 7.5, and a protease inhibitor cocktail (cOmplete^TM^, Roche Diagnostics, Mannheim, Germany). The homogenate was prepared using a glass–Teflon homogenizer. This and all subsequent procedures were carried out at 4 °C. Unbroken cells and nuclei were centrifuged at 600× *g* for 10 min, and the supernatant was centrifuged again at 10,000× *g* for 20 min. The mitochondrial pellet was suspended in the mitochondrial isolation buffer and stored at −80 °C after the determination of protein content [22].

CIII activity was determined using 10–15 µg of crude mitochondria dissolved in phosphate buffer (50 mM potassium phosphate pH 7.8, 0.35% BSA, 2 mM EDTA, 300 µM KCN) under constant agitation at 37 °C, using a dual-wavelength spectrophotometer (V550 Jasco Europe, Modena, Italy). The activity was measured as antimycin A-sensitive ubiquinol–cytochrome *c* reductase activity, in the presence of 50 µM decylbenzoquinol (DBH_2_) and 20 µM bovine heart cytochrome *c* (λ: 550–540 nm; ε_cyt *c*_ = 19.1 mM^−1^ cm^−1^) [21]. CIII redox activity was normalized to protein content [22] and CS activity [20].

### 2.7. Mitochondrial ATP Synthesis

The rate of mitochondrial ATP synthesis was measured in digitonin-permeabilized control and mutant cybrids by using the luciferin/luciferase assay, according to the methods previously described in [23], with minor modifications [24]. Briefly, after trypsinization, cells (10 × 10^6^/mL) were suspended in a buffer containing 150 mM KCl, 25 mM Tris-HCl, 2 mM EDTA, 0.1% BSA, 10 mM potassium phosphate, and 0.1 mM MgCl_2_, pH 7.4, kept at room temperature for 15 min, and then incubated with 50 μg/mL digitonin until 90–100% of cells were positive for erythrosine staining. Aliquots of 3 × 10^5^ permeabilized cells were incubated in the same buffer in the presence of the adenylate kinase inhibitor P1,P5-di(adenosine-5′) pentaphosphate (0.1 mM) and the CI substrates (1 mM malate plus 1 mM pyruvate), the CII substrate (5 mM succinate plus 2 μg/mL rotenone), the CIII substrate (50 µM DBH_2_ plus 1 μM rotenone and 5 mM malonate), or the glycerol-3-phosphate dehydrogenase substrate (20 mM G3P plus 1 μM rotenone and 5 mM malonate). After the addition of 0.1 mM ADP, chemiluminescence was determined as a function of time with a luminometer. The chemiluminescence signal was calibrated with an internal ATP standard after the addition of 10 μM oligomycin. The rates of ATP synthesis were normalized to protein content [22] and CS activity [20].

### 2.8. SDS-PAGE and Western Blotting

Crude mitochondria lysates were prepared using RIPA lysis buffer following the standard protocol, and protein content was measured [22]. First, 40 µg of proteins was separated by 12% sodium dodecyl–polyacrylamide gel electrophoresis (SDS-PAGE) and transferred onto a nitrocellulose membrane (Bio-Rad, Hertfordshire, UK) for Western blotting analysis. The nitrocellulose membranes were incubated overnight at 4 °C with anti-NDUFA9 (1:1000), anti-NDUFB8 (1:1000), anti-NDUFS3 (1:1000), anti-CORE2 (1:1000), anti-COX-IV (1:1000), anti-αATPase (1:1000), and anti-SDHA subunit (1:10,000). Primary antibodies were visualized using appropriate horseradish peroxidase-conjugated secondary antibodies (1:2000). The chemiluminescence signals were revealed using an ECL Western blotting kit (Amersham Bioscience, Buckinghamshire, UK) and measured with the Gel Logic 1500 Imaging System (Kodak, Rochester, NY, USA).

### 2.9. Respiratory Supercomplex Assembly

The supramolecular organization of respiratory complexes was assessed in mitochondria-enriched fractions (mitoplasts) by blue native gel electrophoresis (BN-PAGE) [25]. Briefly, mitoplasts were isolated from cell pellets (~10 × 10^6^ cells) using digitonin (final concentration of 50 μg/mL) and suspended in PBS. Protein concentration was determined [22]. After centrifugation, the pellet was suspended (final concentration 5 mg_protein_/mL) in 150 mM K-acetate, 30 mM HEPES pH 7.4, 10% glycerol, 1 mM PMSF, and 10 mg/mL digitonin (digitonin/protein ratio of 2 g/g) and incubated on ice for 30 min. Samples were centrifuged for 2 min at 600× *g*, and the supernatant, with supercomplexes sample buffer (5% Serva G Blue in 750 mM aminocaproic acid), was loaded onto a 3–12% gradient gel. After electrophoresis, the gels were analyzed by CI In-Gel Activity (CI-IGA) or Western blotting. For CI-IGA, the gels were incubated with 5 mM Tris-HCl (pH 7.4), 0.15 mM NADH, and 2.5 mg/mL MTT at room temperature [25].

## 3. Results

### 3.1. Proband’s Case Report

**Figure 1:**
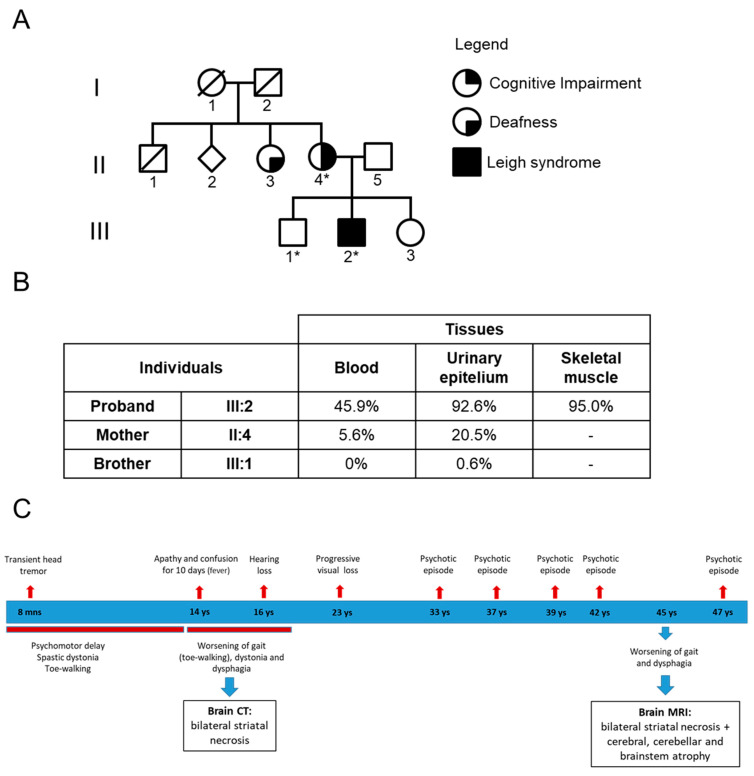
Pedigree and genetic analysis: (**A**) Pedigree of the family. Asterisks indicate the DNA available. (**B**) Heteroplasmy evaluation of the m.15635C>T variant, by NGS approach, in all tissues and family members available. (**C**) Graphical timeline showing the evolution of the symptoms as they appeared throughout the patient’s life.

The proband is a 47-year-old male, the second of three siblings from non-consanguineous parents (individual III-2, Figure 1A). Figure 1C shows a graphical timeline highlighting the evolution of the symptoms as they appeared throughout the patient’s life. He was born after a difficult delivery and started having neurological symptoms aged 8 months, with the appearance of a transient head tremor. He had delayed psychomotor developmental walking at 22 months (toe-walking), with frequent falls, and said his first words at 2 years, presenting speech difficulties for which he started logopedic treatment. His intellectual disability required school support. At age 14, during a febrile episode, he presented with apathy and mental confusion lasting for about 10 days. During adolescence, his gait difficulties worsened, and he also presented exercise intolerance and slowly progressive bilateral deafness.

He was first seen at our institute at age 15. The neurological examination showed facial asymmetry, hypophonia, mild scoliosis, equine feet, mild diffuse muscle hypotrophy, generalized mixed hypertonus with prominent spastic features in the lower limbs, “trochlea dentata” sign in the upper limbs and dystonia of the right side, postural upper limb tremor, bilateral Babinski sign, brisk deep-tendon reflexes, and spastic gait more evident on the right side.

**Figure 2:**
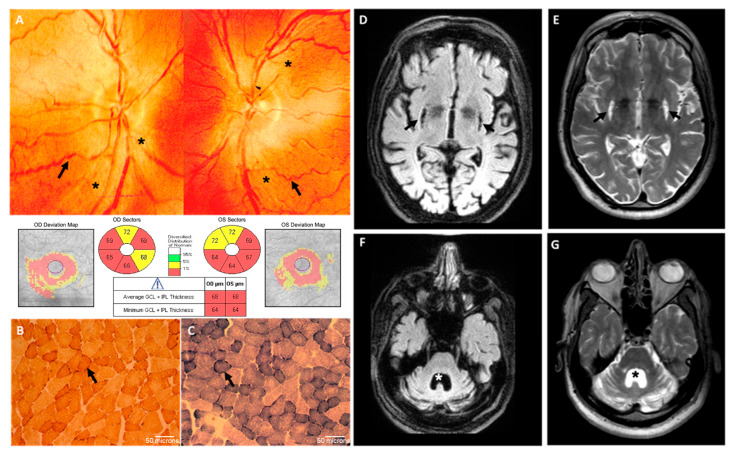
Clinical data of the proband: (**A**) Optical coherence tomography (OCT). The fundus photography images of the patient’s eyes display a moderate swelling of the optic disc fibers (asterisks) without cup and the presence of peripapillary microangiopathy (arrows) (top panel); structural OCT performed in the macular area confirmed a thinning of the GC-IPL in the central area around the fovea (bottom panel); GC-IPL: ganglion cell inner plexiform layers. (**B**,**C**) Muscle biopsy from the proband: histoenzymatic staining for both COX and SDH activities revealed increased subsarcolemmal staining (arrows), indicative of a moderate increase in mitochondrial mass, which is usually a compensatory feature. No COX-deficient or ragged red fibers were observed. (**D**–**G**) Brain MRI: bilateral striatal necrosis (arrows) (**D**,**E**), severe cerebellar atrophy and mild brainstem atrophy with consensual 4th ventricle dilation are evident (asterisk) (**F**,**G**).

Brain CT scan showed symmetric bilateral basal ganglia lesions and mild cerebellar atrophy. EMG was normal, while EEG showed sporadic theta activity. Laboratory exams were normal except for a mild increase in lactic acid levels after standardized exercise (25.3 mg/dL; nv 5–22 mg/dL), which did not promptly recover after 15 min of resting (23 mg/dL). At 16 years of age, he started complaining of dysphagia, and bilateral sensorineural hearing loss was recognized at audiometry. At 23 years, he also showed progressive visual loss. Neuro-ophthalmologic examination showed moderate swelling of the retinal fibers at the optic nerve head and some tortuosity of the peripapillary vessels on fundus picture, whereas optical coherence tomography (OCT) documented loss of retinal ganglion cells in the macula (Figure 2A). Photopic and scotopic ERG showed abnormal bilateral responses. Muscle biopsy performed at 23 years of age showed only some subsarcolemmal increase in cytochrome *c* oxidase (COX) and succinate dehydrogenase (SDH) staining, indicative of moderate proliferation of the mitochondrial mass (Figure 2B,C). Electromyography was normal.

At age 33, he experienced a prolonged episode of psychosis with death delirium and visual hallucinations. Therapy with different neuroleptic drugs (olanzapine, risperidone, quetiapine, chlorpromazine, and promazine) was administered, and he developed a neuroleptic-induced dystonic syndrome after a short period. A therapy with benzodiazepines and trazodone was then started, with an improvement of the psychiatric symptoms. At 33 years, neurological examination also showed axial dystonia and dystonic features of the legs and right foot, reflex myoclonus, mild incoordination, active archaic reflexes, and right digitigrade and spastic gait without arm swing. Neuropsychological evaluation confirmed the presence of moderate intellectual disability (verbal QI: 52).

Similar psychotic episodes recurred at 37, 39, 42, and 47 years of age, successfully managed with different combinations of benzodiazepines, SSRIs, and low doses of neuroleptics (amisulpride and clozapine).

At 45 years, he was admitted again to the hospital for a global worsening of the neurological picture and specifically of the gait ataxia and dysphagia, with frequent episodes of vomiting. Neurological examination showed a severe dysarthria, mirror movements in the upper limbs, diffuse mixed hypertonus in all four limbs, dystonic posture of the lower limbs more evident on the right leg with equinus foot, archaic reflexes, and dystonic–spastic and apraxic gait (R > L).

Brain MRI confirmed the presence of bilateral striatal necrosis and severe cerebellar and mild brainstem atrophy (Figure 2D–G). DaT-SCAN imaging showed normal pre-synaptic uptake of the dopamine transporter tracer at the level of the caudate and proximal putamen, whereas this uptake was absent at the level of the medium–distal putamen bilaterally. Cardiological evaluation showed left ventricular non-obstructive hypertrophy and right bundle conduction defect.

### 3.2. Family History

The patient’s maternal grandmother died at 28 years of age after pleuritis; she had four children, one of whom (male) died aged 1 month (he also had bone abnormalities), while another (female, 66 years) has hearing difficulties (Figure 1A).

The patient’s mother is an 81-year-old-woman. By age 25, she complained of mild stypsis and diffuse joint pain. Her neurological complaints began at age 57, after some episodes of positional vertigo and evidence of mild deafness; then, at 59 years, she presented mild cognitive impairment. She was referred to our institute at 61 years, where the neurological examination revealed the presence of right Lasegue sign, archaic reflexes, and a positive Romberg sign. The laboratory exams showed normal lactic acid levels after standardized exercise and glucose intolerance. Audiometry disclosed a mild unilateral sensorineural deafness and brainstem auditory evoked potentials showed a bilateral symmetric increase in the central conduction of the auditory pathway. Brain MRI was unremarkable, and neuropsychological evaluation showed two single deficits in semantic fluency and selective visual attention tasks.

### 3.3. Genetic Findings

The complete mtDNA sequence analysis performed in the proband’s blood cells revealed a heteroplasmic variant affecting position m.15635T>C in the *MT-CYB* gene, leading to the amino acid change p.Ser297Pro (GenBank ID #AY882394.1 and published in [26]). This variant was previously reported in a patient with a multisystemic mitochondrial disorder [16] and is classified as Variant of Uncertain Significance (VUS) according to the ACMG criteria, but the non-conservative amino acid change, the highly conserved position (Ser297), the heteroplasmy, and the recurrence in another patient affected by mitochondrial disease strongly suggests its pathogenic potential in accordance with recently defined criteria for mtDNA mutations [27,28]. Furthermore, while this variant has been reported only twice, a third case was reported with another variant affecting the same position (m. 15635T>G) but leading to a different amino acid change (p.Ser297Ala) (https://www.mitomap.org/MITOMAP (accessed on 22 January 2025)). According to the array of haplogroup-specific variants, the mtDNA of this patient belongs to haplogroup K1c. Noticeably, other haplogroup-specific polymorphic variants co-existed in the *MT-CYB* gene of the proband; while most of them are common to the majority of European haplogroups, m.14798T>C (p.Phe18Leu) is specific to haplogroup K, and m.14757T>C (p.Met4Thr) is private, both homoplasmic (see Supplementary Figure S1).

The heteroplasmy of the m.15635T>C variant varied in different tissues of the proband, being 45.9% in blood cells, 92.6% in the urinary sediment, and 95.0% in skeletal muscle (Figure 1B). The variant was absent in the brother’s blood cells and at a very low level (0.6%) in urinary sediment, whereas the proband’s mother had lower variant loads in blood cells (5.6%) and urinary sediment (20.5%). The mean coverage at the position m.15635 was 1748x (min 897x–max 2387x–median 1814x).

### 3.4. Cybrid Studies

To conclusively assess the pathogenicity of the m.15635T>C (p.Ser297Pro) variant in the *MT-CYB* gene, we generated cybrids from the fibroblast cell line obtained from the mother’ skin biopsy (50% mutant load) after informed consent and IRB approval. We were able to select two syngeneic cybrid clones: one homoplasmic wild-type (here used as a control) and one homoplasmic mutant.

**Figure 3:**
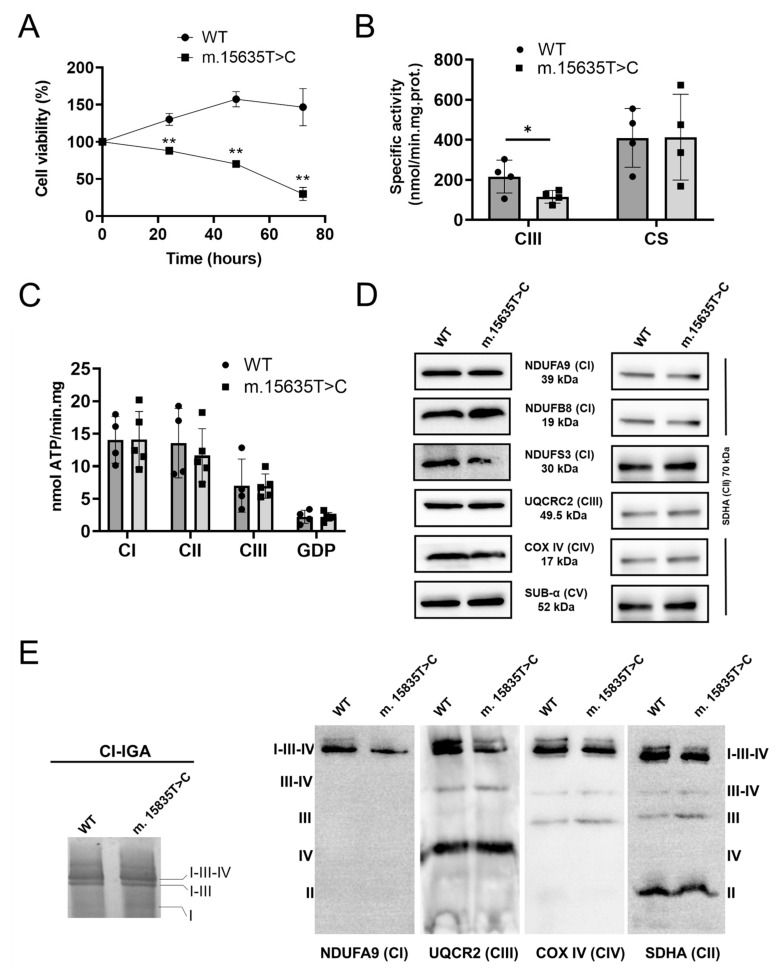
Biochemical studies on cybrids: (**A**) Time course of cell viability in glucose-free medium supplemented with galactose. Two syngeneic cybrid clones, 100% wild-type and bearing the homoplasmic m.15635T>C (p.297S>P) variant, were used. The SRB absorbance value at time zero corresponds to 100% viable cells. Each data point represents the mean ± SD of at least three experiments. Statistical analysis was performed by *t*-test; ** *p* < 0.01. (**B**) Respiratory complex III and citrate synthase activities were assessed as described in [21]. Data are the means ± SD of at least three experiments. Statistical analysis was performed by *t*-test; * *p* < 0.05. (**C**) The rate of ATP synthesis driven by CI (malate/pyruvate), CII (succinate), CIII (DBH_2_), and glycerol-3-phosphate dehydrogenase (G3P) was measured as previously described in [21]. Data are the means ± SD of at least four experiments. (**D**) SDS-PAGE and Western blot analyses of representative respiratory chain subunits from 40 μg of crude mitochondria were performed as previously reported [21]. Western blot analysis was carried out using the antibodies against the CI (NDUFA9, NDUFS3, and NDUFB8) subunits, the CII-SDHA subunit, the CIII-UQCRC2 subunit, the CIV-COX IV subunit, and the CV-*α* subunit. (**E**) Digitonin-solubilized (2 g/g protein) mitoplasts, isolated from WT and homoplasmic mutated cybrids, were separated by BN-PAGE. One gel was used for the CI In-Gel Activity (CI-IGA), as described in the Materials and Methods. The other gels were transferred to nitrocellulose membranes for Western blot analysis, using antibodies against the indicated subunits of OXPHOS complexes.

To evaluate mitochondrial function, the cybrid clones were grown in glucose-free DMEM (DMEM-galactose), which forces cells to rely on OXPHOS for ATP production [29]. Figure 3A shows that the viability of the homoplasmic mutant clone decreased significantly compared to the wild-type after 24–48 h of DMEM–galactose incubation. Furthermore, the homoplasmic mutant cybrids showed a dramatic loss of viability compared to wild-type syngeneic cybrids after 72 h of incubation in DMEM–galactose. These results suggest that the m.15635T>C variant affects mitochondrial efficiency.

To investigate the biochemical effects of the *MT-CYB* variant on the mitochondrial respiratory chain, we measured the redox activity of complex III in crude mitochondria [21]. Cells carrying the homoplasmic m.15635T>C variant showed a significant decrease in CIII activity (approximately 50% lower) compared to wild-type syngeneic cells, indicating that the variant affected CIII redox activity (Figure 3B).

Despite the defect in mitochondrial efficiency and the decrease in CIII redox activity, the rates of mitochondrial ATP synthesis in digitonin-permeabilized cybrids driven by CI (pyruvate/malate), CII (succinate), CIII (decylbenzoquinol, DBH_2_), and glycerol-3-phosphate dehydrogenase (glycerol-3-phosphate, G3P) substrates were not different between mutant and control cells, as shown in Figure 3C.

We then inquired as to whether the m.15635T>C variant alters the steady-state expression levels of respiratory complex subunits. As shown in Figure 3D, the levels of representative subunits of different complexes analyzed were similar in control and mutant cybrids when their ratios to SDHA (CII) were compared.

Additionally, supramolecular organization of respiratory supercomplexes was assessed by CI In-Gel Activity (CI-IGA) and Western blotting after blue native gel electrophoresis (BN-PAGE) in the presence of a high concentration of the mild detergent digitonin, which maintains loose interactions between complexes [25]. No appreciable difference in cybrids bearing the m.15635T>C homoplasmic variant compared to wild-type syngeneic cybrids was observed (Figure 3E). Taken together, these results suggest that the m.15635T>C variant affects the CIII activity but not the CIII steady-state levels or the complex/supercomplex assembly.

### 3.5. Structural Analysis

**Figure 4:**
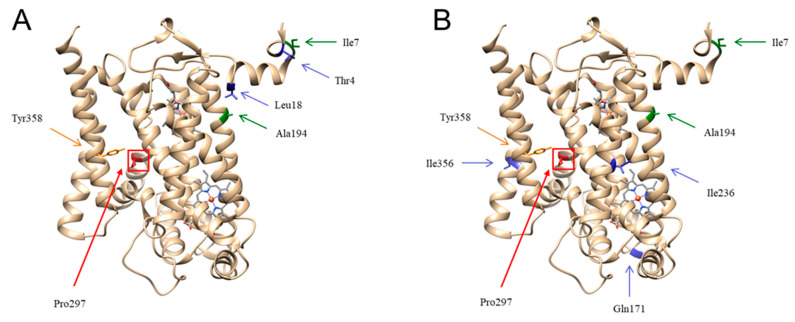
In silico analysis of human monomeric mutated cytochrome *b* in different haplogroup backgrounds: The highly conserved amino acid positions Pro297 and Tyr358 are indicated in red and orange, respectively. Different missense variants of haplogroup K1c (**A**) and haplogroup J2b (**B**) are indicated by the green and blue arrows. The two missense variants common to the two haplogroups in MT-CYB (Ile7 and Ala194) are highlighted in green, while the other variants carried specifically by each haplogroup are shown in blue.

The biochemical results obtained in the cybrid studies showed a milder phenotype compared to that previously reported in the very same cellular model by Fragaky et al. [16]. Consequently, we investigated in silico the potential involvement of missense variants associated with the two mtDNA backgrounds for which the cybrids of the two patients differed. The proband described in this study harbored the K1c haplogroup, while the previously reported patient carried the J2b haplogroup [16]. Figure 4 shows the structure of human cytochrome *b*, in which the amino acid substitution p.Ser297Pro is highlighted (in red) together with the different variants of the two haplogroups. These two haplogroups share two common missense variants in MT-CYB (m.14766C>T p.Thr7Ile and m.15326A>G p.T194A, in green), but they also have some haplogroup-specific missense variants (in blue). Haplogroup K1c carries the specific variant of haplogroup K (m.14798 T>C p.Phe18Leu) but also a private variant at position m.14757T>C p.Met4Thr (Figure 4A), while haplogroup J2b has three more missense variants (m.15257G>A p.Asp171Gln, m.15452C>A p.Leu236Ile, and m.15812G>A p.Val356Ile) (Figure 4B). Structural analysis revealed that none of the haplogroup missense variants interact directly with Pro297, but in haplogroup J2b, Ile356 is close to Tyr358, which has previously been suggested to form a H bond with Ser297 in the wild-type that could be lost in mutants [16].

## 4. Discussion

In this report, we describe the second case of a patient with a mitochondrial disease phenotype associated with the ultra-rare heteroplasmic *MT-CYB* gene variant affecting position m.15635T>C, which obeys the criteria for being pathogenic. Our cybrid investigations certified the co-segregation with this variant of a reduced complex III activity (50% reduction in the homoplasmic clone) and impaired cell viability in galactose medium when cells are forced to rely on OXPHOS. Thus, this variant should now be considered pathogenic, associated with either severe multisystemic disease or Leigh syndrome.

On the clinical level, our long-surviving patient with Leigh syndrome differs in severity from the previously described patient, a very severe pediatric case undergoing death 24 h after birth. Our patient had a fairly classical course of Leigh syndrome with characteristic bilateral lesions in the basal ganglia, clinically associated with spastic dystonia, loss of retinal ganglion cells, sensorineural hearing loss, and recurrence of psychotic episodes. Certainly, the possibility of slight differences in the heteroplasmic load of the m.15635T>C variant in diverse tissues and organs in our patient might partially explain the dramatic clinical discrepancy between the only two reported patients to date, as the pediatric case exhibited homoplasmic mutant mtDNA in all investigated tissues. Our proband’s mother carried an intermediate heteroplasmic load of the m.15635T>C variant, displaying some late-onset neurological disturbances such as hearing loss and mild cognitive impairment. Additionally, along the maternal lineage, one uncle died shortly after birth, with reported bone abnormalities, and one aunt suffered some hearing loss. Although genetic analysis is unavailable for these family members, we can speculate that different heteroplasmic loads of the same m.15635T>C variant might underlie these distinct phenotypes.

On the genetic level, the m.15635T>C variant of mtDNA, leading to the p.Ser297Pro amino acid change in a highly conserved position, has been reported only twice, in these two patients. In a previous report by Achilli et al. [26], the full mtDNA sequence of our patient was in fact published in a population genetic study and, thus, should not be considered as an independent case. Furthermore, a previous report described a different nucleotide change affecting the same amino acid position (m.15635T>G) but leading to a different amino acid change (p.Ser297Ala). This latter substitution is conservative compared to the more drastic p.Ser297Pro change. The m.15635T>G variant was found in a family with Leber hereditary optic neuropathy (LHON) associated with the m.11778G>A primary mutation and was speculated to be a possible disease modifier [30]. In conclusion, the only two cases associated with the m.15635T>C variant are unequivocally associated with patients affected by severe phenotypes of mitochondrial disease. The phenotypic differences between these two cases may be attributable to their distinct mtDNA haplogroup backgrounds: J2b versus K1c. Haplogroup J2b harbors two missense variants in *MT-CYB*: m.15257G>A and m.15812G>A, both previously discussed as relevant in Leber hereditary optic neuropathy (LHON), potentially acting as modifiers increasing the penetrance in families carrying the m.11778G>A and m.14484T>C variants [31,32]. Noticeably, the structural analysis performed on the cytochrome *b* structure showed that one of the specific haplogroup Jb2 missense variants (m.15812G>A p.Val356Ile) lies close to the highly conserved Tyr358 residue that has been suggested to possibly form a H bond together with the Ser297 eventually lost in the mutant protein, ultimately reducing its stability [16]. We can speculate that the substitution of Val356 with an amino acid with greater steric hindrance, such as Ile, may affect the protein stability, worsening the pathological effect of Ser297Pro mutation. Conversely, haplogroup K1c had a different array of *MT-CYB* variants that did not reside close to the 297 or 358 positions, suggesting a reduced impact of this haplogroup on the pathogenic mutation. Furthermore, a recent study investigating metabolic differences amongst mtDNA haplogroups documented that haplogroup J was the most sensitive to rotenone, while haplogroup K demonstrated a capacity to orchestrate an efficient compensatory strategy, specifically by activating mitochondrial biogenesis [33]. The different behaviors of the two mtDNA backgrounds and related intragenic *MT-CYB* variants may add an additional layer as a modifying factor impacting the distinct phenotypes of the two reported cases carrying the m.15635T>G pathogenic variant.

For both patients, functional studies were performed using cybrids harboring homoplasmic mutant loads, again leading to some remarkable differences that need to be commented on. Cybrids generated from our patient showed complex III redox activity reduced by approximately 50%, without affecting complex III assembly and, ultimately, ATP synthesis. Cybrids from the pediatric case displayed a drastic reduction in complex III assembly (mirroring the findings observed in the liver), unfortunately without providing the complex I assembly assessment. Furthermore, the polarographic analysis showed a profound decrease in oxidation from different entry points within the respiratory chain. The complex III redox activity of the pediatric m.15635T>C variant was assessed only in tissues and fibroblasts, with variable reductions ranging from 17% in the liver to 82% in fibroblasts, but remarkably normal activity in skeletal muscle. Thus, also on the functional level, our proband displayed a milder functional defect compatible with the milder clinical phenotype, whereas the pediatric case, investigated with assays that were not directly comparable, showed a strikingly more severe OXPHOS dysfunction.

In conclusion, the biochemical differences between our case and the previously published pediatric case are essentially congruent with their respective phenotypes. The different magnitudes of OXPHOS defects observed in the cybrid studies carrying the equally homoplasmic m.15635T>C variant on two distinguished mtDNA haplogroup backgrounds (J vs. K), carrying different sets of intragenic *MT-CYB* variants, highlights this as the only difference observable in cybrids. Thus, the current findings point to a modifying impact of mtDNA sequence background on the overall phenotype. We propose that this *MT-CYB* m.15635T>C variant has an undisputable pathogenic potential, which also seems to be highly modulated by the genetic background of mtDNA and the intrinsic *MT-CYB* gene sequence variability. This rare mtDNA variant should now be considered to be pathogenic, with variable phenotypic expression.

## Data Availability

Data is contained within the article and Supplementary Material.

## Supplementary Materials

The following supporting information can be downloaded at: https://www.mdpi.com/article/10.3390/ijms26031116/s1.

## References

[1] Lake N.J., Bird M.J., Isohanni P., Paetau A. (2015). Leigh Syndrome: Neuropathology and Pathogenesis. J. Neuropathol. Exp. Neurol..

[2] Ruhoy I.S., Saneto R.P. (2014). The Genetics of Leigh Syndrome and Its Implications for Clinical Practice and Risk Management. Appl. Clin. Genet..

[3] Rahman S. (2023). Leigh Syndrome. Handb. Clin. Neurol..

[4] Carelli V., Chan D.C. (2014). Mitochondrial DNA: Impacting Central and Peripheral Nervous Systems. Neuron.

[5] Fernández-Vizarra E., Zeviani M. (2015). Nuclear Gene Mutations as the Cause of Mitochondrial Complex III Deficiency. Front. Genet..

[6] Bénit P., Lebon S., Rustin P. (2009). Respiratory-Chain Diseases Related to Complex III Deficiency. Biochim. Biophys. Acta.

[7] Rugolo M., Zanna C., Ghelli A.M. (2021). Organization of the Respiratory Supercomplexes in Cells with Defective Complex III: Structural Features and Metabolic Consequences. Life.

[8] Andreu A.L., Hanna M.G., Reichmann H., Bruno C., Penn A.S., Tanji K., Pallotti F., Iwata S., Bonilla E., Lach B. (1999). Exercise Intolerance Due to Mutations in the Cytochrome b Gene of Mitochondrial DNA. N. Engl. J. Med..

[9] Carossa V., Ghelli A., Tropeano C.V., Valentino M.L., Iommarini L., Maresca A., Caporali L., La Morgia C., Liguori R., Barboni P. (2014). A Novel In-Frame 18-Bp Microdeletion in MT-CYB Causes a Multisystem Disorder with Prominent Exercise Intolerance. Hum. Mutat..

[10] De Coo I.F., Renier W.O., Ruitenbeek W., Ter Laak H.J., Bakker M., Schägger H., Van Oost B.A., Smeets H.J. (1999). A 4-Base Pair Deletion in the Mitochondrial Cytochrome b Gene Associated with Parkinsonism/MELAS Overlap Syndrome. Ann. Neurol..

[11] Rana M., de Coo I., Diaz F., Smeets H., Moraes C.T. (2000). An Out-of-Frame Cytochrome b Gene Deletion from a Patient with Parkinsonism Is Associated with Impaired Complex III Assembly and an Increase in Free Radical Production. Ann. Neurol..

[12] Protasoni M., Pérez-Pérez R., Lobo-Jarne T., Harbour M.E., Ding S., Peñas A., Diaz F., Moraes C.T., Fearnley I.M., Zeviani M. (2020). Respiratory Supercomplexes Act as a Platform for Complex III-Mediated Maturation of Human Mitochondrial Complexes I and IV. EMBO J..

[13] Wibrand F., Ravn K., Schwartz M., Rosenberg T., Horn N., Vissing J. (2001). Multisystem Disorder Associated with a Missense Mutation in the Mitochondrial Cytochrome b Gene. Ann. Neurol..

[14] Emmanuele V., Sotiriou E., Rios P.G., Ganesh J., Ichord R., Foley A.R., Akman H.O., Dimauro S. (2013). A Novel Mutation in the Mitochondrial DNA Cytochrome b Gene (MTCYB) in a Patient with Mitochondrial Encephalomyopathy, Lactic Acidosis, and Strokelike Episodes Syndrome. J. Child. Neurol..

[15] Mancuso M., Nesti C., Ienco E.C., Orsucci D., Pizzanelli C., Chiti A., Giorgi F.S., Meschini M.C., Fontanini G., Santorelli F.M. (2014). Novel MTCYB Mutation in a Young Patient with Recurrent Stroke-like Episodes and Status Epilepticus. Am. J. Med. Genet. A.

[16] Fragaki K., Procaccio V., Bannwarth S., Serre V., O’Hearn S., Potluri P., Augé G., Casagrande F., Caruba C., Lambert J.C. (2009). A Neonatal Polyvisceral Failure Linked to a de Novo Homoplasmic Mutation in the Mitochondrially Encoded Cytochrome b Gene. Mitochondrion.

[17] Achilli A., Iommarini L., Olivieri A., Pala M., Hooshiar Kashani B., Reynier P., La Morgia C., Valentino M.L., Liguori R., Pizza F. (2012). Rare Primary Mitochondrial DNA Mutations and Probable Synergistic Variants in Leber’s Hereditary Optic Neuropathy. PLoS ONE.

[18] King M.P., Attadi G. (1996). Mitochondria-Mediated Transformation of Human Rho(0) Cells. Meth. Enzymol..

[19] Scarlatti F., Sala G., Somenzi G., Signorelli P., Sacchi N., Ghidoni R. (2003). Resveratrol Induces Growth Inhibition and Apoptosis in Metastatic Breast Cancer Cells via de Novo Ceramide Signaling. FASEB J..

[20] Trounce I.A., Kim Y.L., Jun A.S., Wallace D.C. (1996). Assessment of Mitochondrial Oxidative Phosphorylation in Patient Muscle Biopsies, Lymphoblasts, and Transmitochondrial Cell Lines. Meth. Enzymol..

[21] Ghelli A., Tropeano C.V., Calvaruso M.A., Marchesini A., Iommarini L., Porcelli A.M., Zanna C., De Nardo V., Martinuzzi A., Wibrand F. (2013). The Cytochrome *b* p.278Y>C Mutation Causative of a Multisystem Disorder Enhances Superoxide Production and Alters Supramolecular Interactions of Respiratory Chain Complexes. Hum. Mol. Genet..

[22] Bradford M.M. (1976). A Rapid and Sensitive Method for the Quantitation of Microgram Quantities of Protein Utilizing the Principle of Protein-Dye Binding. Anal. Biochem..

[23] Manfredi G., Yang L., Gajewski C.D., Mattiazzi M. (2002). Measurements of ATP in Mammalian Cells. Methods.

[24] Giorgio V., Petronilli V., Ghelli A., Carelli V., Rugolo M., Lenaz G., Bernardi P. (2012). The Effects of Idebenone on Mitochondrial Bioenergetics. Biochim. Biophys. Acta.

[25] Tropeano C.V., Aleo S.J., Zanna C., Roberti M., Scandiffio L., Loguercio Polosa P., Fiori J., Porru E., Roda A., Carelli V. (2020). Fine-Tuning of the Respiratory Complexes Stability and Supercomplexes Assembly in Cells Defective of Complex III. Biochim. Biophys. Acta. Bioenerg..

[26] Achilli A., Rengo C., Battaglia V., Pala M., Olivieri A., Fornarino S., Magri C., Scozzari R., Babudri N., Santachiara-Benerecetti A.S. (2005). Saami and Berbers--an Unexpected Mitochondrial DNA Link. Am. J. Hum. Genet..

[27] Montoya J., López-Gallardo E., Díez-Sánchez C., López-Pérez M.J., Ruiz-Pesini E. (2009). 20 Years of Human mtDNA Pathologic Point Mutations: Carefully Reading the Pathogenicity Criteria. Biochim. Biophys. Acta.

[28] McCormick E.M., Lott M.T., Dulik M.C., Shen L., Attimonelli M., Vitale O., Karaa A., Bai R., Pineda-Alvarez D.E., Singh L.N. (2020). Specifications of the ACMG/AMP Standards and Guidelines for Mitochondrial DNA Variant Interpretation. Hum. Mutat..

[29] Robinson B.H. (1996). Use of Fibroblast and Lymphoblast Cultures for Detection of Respiratory Chain Defects. Meth. Enzymol..

[30] Qu J., Wang Y., Tong Y., Zhou X., Zhao F., Yang L., Zhang S., Zhang J., West C.E., Guan M.-X. (2010). Leber’s Hereditary Optic Neuropathy Affects Only Female Matrilineal Relatives in Two Chinese Families. Invest. Ophthalmol. Vis. Sci..

[31] Carelli V., Achilli A., Valentino M.L., Rengo C., Semino O., Pala M., Olivieri A., Mattiazzi M., Pallotti F., Carrara F. (2006). Haplogroup Effects and Recombination of Mitochondrial DNA: Novel Clues from the Analysis of Leber Hereditary Optic Neuropathy Pedigrees. Am. J. Hum. Genet..

[32] Hudson G., Carelli V., Spruijt L., Gerards M., Mowbray C., Achilli A., Pyle A., Elson J., Howell N., La Morgia C. (2007). Clinical Expression of Leber Hereditary Optic Neuropathy Is Affected by the Mitochondrial DNA–Haplogroup Background. Am. J. Hum. Genet..

[33] Strobbe D., Caporali L., Iommarini L., Maresca A., Montopoli M., Martinuzzi A., Achilli A., Olivieri A., Torroni A., Carelli V. (2018). Haplogroup J Mitogenomes Are the Most Sensitive to the Pesticide Rotenone: Relevance for Human Diseases. Neurobiol. Dis..

