# Supplementary material for: Adult Leigh Syndrome Associated with the m.15635T>C Mitochondrial DNA Variant Affecting the Cytochrome b (MT-CYB) Gene
Source: Int J Mol Sci. 2025 Jan 27;26(3):1116. doi: 10.3390/ijms26031116 (PMC11817157; doi:10.3390/ijms26031116)
Supplement: Supplementary file 1 [file ijms-26-01116-s001.zip › ijms-3354304-supplementary.pdf]

Figure S1

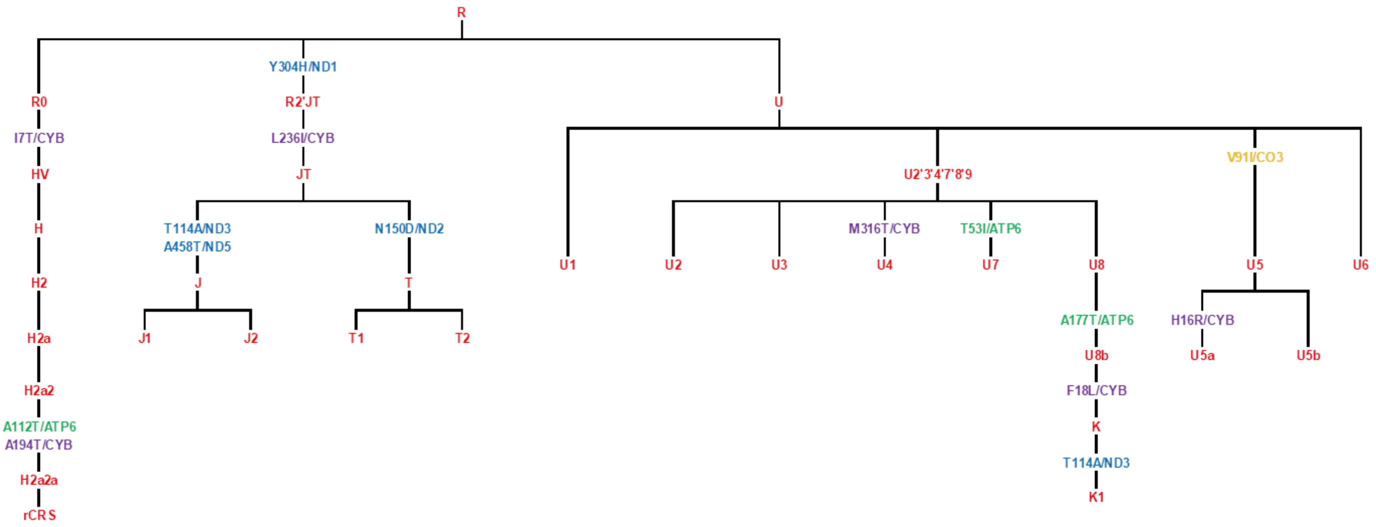

Figure S1: Schematic phylogenetic tree of mtDNA haplogroups (https://www.phylotree.org/) with missensese variants highlighted (blue in Complex I, purple in Complex III, yellow in Complex IV, green in Complex V) and mtDNA haplogroup clades (in red).
